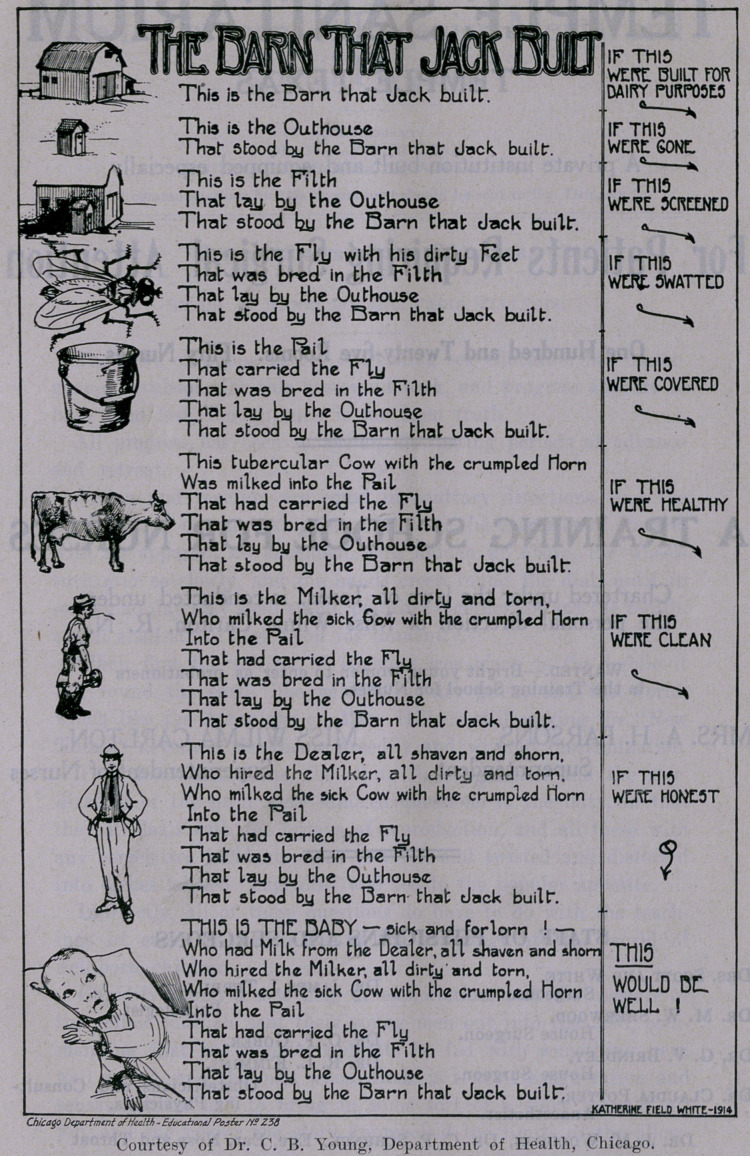# Some Recent Observations on Pyorrhea Alveolaris, or Rigg’s Disease

**Published:** 1914-12

**Authors:** C. C. Bass

**Affiliations:** Tulane College of Medicine, New Orleans, Louisiana


### Some Recent Observations on Pyorrhea Alveolaris, or Rigg's Disease.

BY C. C. BASS, M. D., TULANE COLLEGE OF MEDICINE, NEW ORLEANS, LOUISIANA.

New interest in this disease has recently developed as a result of the announcement by Smith and Barrett of the causative relation that entameba buccalis bears to the disease and the further observation that rapid improvement results from treatment with emetine hydrochloride. Though amebae and other protozoae have been known for a long time to inhabit the mouth, teeth, and gums, their pathogenicity seems not to have previously been seriously considered. Since the observations of Barrett and Smith, Dr. F. M. Johns and I have studied the disease considerably with special reference to the presence of amebae, and the influence of treatment with emetine and ipecac on the amebae and on the disease process.

Although our studies are very incomplete, some observations seem of so much importance that I am pleased to bring them to your attention today. Barrett and Smith found amebae present in all of their cases of the disease. Chiavaro found them present in twenty-two cases of pyorrhea and in the mouths of fourteen other people not supposed to have the disease. He concluded that "they are present in the lesions in all cases of pyorrhea and that they are non-pathogenic, but on the other hand probably assist in the auto-sterilization of the gums by destroying bacteria."

We have found amebae in the lesions of more than 130 patients having pyorrhea alveolaris in stages varying from the earliest to the latest. We have failed to find them in three instances where we made not very thorough examinations of cases we believed to have Rigg's disease. It is not my intention to report here in detail our studies, but rather to mention some of the indications of the observations thus far made.

The amebae are easily demonstrated in either fresh or stained specimens. The pus or scrapings from the depth of the lesion should be diluted with a little water or salt solution on a slide, covered with a cover glass and examined. Motile amebae from about eight to about thirty microns in diameter are usually readily found. An excellent stain is carbol fuchsin one-fourth minute, followed by Loeffler's methylene blue one-half minute. In stained specimens dark stained objects, apparently the nuclei of pus cells, can be seen inside the parasites.

Those who have not had occasion to pay special attention to this disease are likely not to fully appreciate its very great prevalence and the course of the disease process. The infection probably never attacks a perfectly normal tooth and gum. Trauma, by which the gum is torn away from the tooth and where food particles or other foreign material prevents prompt healing, offers favorable soil for establishing the infection. Such damage is especially likely to result in the inter-dental gum from the use of tooth picks, etc., and healing is likely to be prevented by food forced between the teeth. Accumulation of tartar on the teeth also makes pressure on the gums and furnishes favorable soil for the infection by amebae that may chance to be introduced from the mouths of others. Infection once established in one or more places, a constant source of supply of amebae is established in the patient's own mouth to infect other gums, whenever damaged so as to permit the amebae to attack them. On account of the burrowing or excavating habits of the amebae, they work in the bottom of the lesion, spreading infection of the secondary bacteria, perhaps attacking the tissues themselves, and thus prevent healing. The process slowly extends deeper and deeper, the peri-dental membrane being destroyed by the long-continued process. Finally the disease reaches the alveolar structure and infection with amebae and accompanying bacteria sooner or later cause suppuration and destruction of not only the peri-dental membrane, but the bony structure as well. The tooth gets loose in its socket and pressure causes pus to flow freely from around the root. Ultimately the suppurating process entirely separates the tooth from its attachment and it is lost. Amebae can be found in pus from gums in all stages of the disease.

In the early stage the diseased gums bleed freely from picking, brushing or sucking them. As the disease extends the gum is dissected away from the tooth, the alveolar process is destroyed and the gum retracts. It is usually this final stage that the patient recognizes as pyorrhea, or Rigg's disease, and the early stage is not recognized.

Smith and Barrett treated with favorable results a large number of cases by injecting a one-half per cent solution of emetine hydrochloride into the pus cavities around the affected teeth. Our experiments have been with emetine given hypodermically. It is needless to describe the many different plans tried. In more than 100 cases treated we have found amebae constantly absent from the lesions in all except two cases after the patient had been given one-half grain emetine hydrochloride hypodermically daily for three successive days. The tendency to bleed stops in twenty-four to forty-eight hours, and where only the soft tissue is involved the red, inflamed gums often appear practically normal in from three to ten days—apparently as quickly as Nature can heal them. Where the bony structure is involved and the teeth are loose there is also rapid improvement and relief from soreness and pain. The pus decreases and loose teeth often get firmer in a few days, but it must be remembered that in most cases where the disease has extended this far the peri-dental membrane is destroyed to a great extent, often almost to the end of the root. Nature cannot grow new peri-dental membrane and retraction must therefore take place to the level of the living membrane. The healing process can be very much hastened by dental treatment, such as scaling, scraping, cleaning, and removing overhanging tissue. It must not be expected that removal of the specific cause and the best dental treatment can save teeth denuded of peri-dental membrane to the very end of the root and hanging in a suppurating cavity or tooth socket.

Once disinfected, the chances of reinfection before healing has taken place or the escape of some amebae make it very desirable to continue treatment for some time. In fact, on account of the general prevalence of the disease it would seem necessary for all persons to continue prophylactic treatment indefinitely. We have experimented considerably to ascertain some practical prophylactic treatment—one that could be used by all. To our great surprise and gratification we find that patients can cure the disease in its early stage by cleanliness and by placing one or two drops of fluid extract of ipecac on the brush before brushing the teeth, and forcing some of the solution of ipecac that is thus made in the mouth, between the teeth. In several instances the amebae disappeared, bleeding stopped, and the gum took on a normal appearance in a few days.

The treatment that now seems most effectual is (1) emetine hydrochloride one-half grain hypodermically daily for three successive days; (2) indicated dental treatment; (3) use of one or two drops of fluid extract of ipecac on the tooth brush once a day indefinitely. We believe now that the latter will prevent the disease, cure mild cases, and should be used by all persons, whether they can have advantage of the emetine and dental treatment or not.

---

District Judge John M. Conley and Attorney W. M. Crook were the principal speakers to address the Jefferson County Medical Society at the regular monthly meeting, which was held November 2d.

That 95 per cent of the criminals are physically defective in some manner and that physicians and surgeons could play a prominent part in the prevention of crime were points brought out by Judge Conley in his address on "The Doctor as a Witness."

Mr. Crook emphasized the fact that a doctor is one whom everyone loves, owing to the invaluable service he performs, declaring that the relationship of a lawyer and his client ends when the case is settled, but with the doctor it is different. His relation with his client or patient continues through life.

A. F. Jatho, another attorney, and several doctors made short talks, general discussions following each.

## Figures and Tables

**Figure f1:**